# An augmented prescribed exercise program (APEP) to improve mobility of older acute medical patients – a randomized, controlled pilot and feasibility trial

**DOI:** 10.1186/s12877-019-1246-4

**Published:** 2019-08-30

**Authors:** Tobias Braun, Christian Grüneberg, Kirsten Süßmilch, Max Wiessmeier, Isabel Schwenk, Sarah Eggert, Annika Machleit-Ebner, Irene Harras, Christian Thiel

**Affiliations:** 1 Department of Applied Health Sciences, Division of Physiotherapy, Hochschule für Gesundheit (University of Applied Sciences), Gesundheitscampus 6-8, 44801 Bochum, Germany; 2 Evangelische Krankenhausgemeinschaft Herne/Castrop-Rauxel gGmbH, Division of Physiotherapy, Castrop-Rauxel, Germany; 3 Evangelische Krankenhausgemeinschaft Herne/Castrop-Rauxel gGmbH, Clinical Trials Center, Wiescherstraße 24, 44623 Herne, Germany; 4 Evangelische Krankenhausgemeinschaft Herne/Castrop-Rauxel gGmbH, Therapeutic management, Wiescherstraße 24, 44623 Herne, Germany; 5 Ruhr-University Bochum, Faculty of Sports Science, Training and Exercise Science, Bochum, Germany

**Keywords:** Mobility limitation, Rehabilitation, Physiotherapy, Exercise therapy, Hospitalization

## Abstract

**Background:**

There is inconclusive evidence for the effectiveness of additional exercise in older hospital patients. The aims of this study were (1) to assess the feasibility of an augmented prescribed exercise program (APEP) in older acute medical patients and (2) to measure the potential effects of APEP on mobility capacity in order to assess the feasibility of a large full-scale study.

**Methods:**

We conducted a single-center, prospective, parallel-group, single-blinded, randomized (1:1) controlled pilot and feasibility trial. Participants were recruited from acute geriatric wards of a general hospital. Key inclusion criteria were: age ≥ 65 years and walking ability. Key exclusion criteria were severe cognitive impairment and medical restriction for physical exercise interventions.

Both groups received usual care, including physiotherapy. Intervention group participants were scheduled for additional exercise sessions (20–30 min, 4-5x/week). Feasibility of the trial design was assessed along pre-defined criteria for process, resources and management. Feasibility of the APEP intervention was analyzed by means of adherence, compliance and safety. Outcomes were measured at baseline and prior to hospital discharge. The primary outcome was mobility capacity (de Morton Mobility Index; DEMMI). Secondary outcomes were walking ability, physical endurance, fear of falling, frailty and length of stay.

**Results:**

Thirty-five participants were recruited (recruitment rate 20.3%). We lost 7 participants to follow-up (retention rate: 80%). Intervention group participants (n = 17) each participated in 5.3 ± 2.2 additional exercise sessions (mean duration: 23.2 ± 4.0 min; mean adherence rate 78% ± 26%). No severe adverse events occurred during study assessments or APEP sessions.

There were no statistically significant differences in mean change scores in any outcome measure. A sample of 124 participants would be required to detect a difference of 4 DEMMI points (ES = 0.45) with a power of 80%.

**Conclusions:**

This small feasibility RCT indicates that an APEP intervention may be safe and feasible in older acute medical patients. APEP may possibly induce small to moderate effects on mobility, but the clinical relevance of these effects may be limited. These results inform the planning of a larger-scale phase III study.

**Trial registration:**

German Clinical Trials Register (DRKS00011262). Registered 27 October 2016.

**Electronic supplementary material:**

The online version of this article (10.1186/s12877-019-1246-4) contains supplementary material, which is available to authorized users.

## Background

Many older hospital patients have a significantly reduced mobility capacity and show low levels of physical activity. There is evidence from cohort and observational studies that 30 to 60% of older medical patients are not able to stand and walk without physical assistance at hospital admission [1–6]. For example, Ostir et al. [5] reported that 78% of older acute hospital patients were either not able to ambulate (36%), or walked with a reduced gait speed of < 0.60 m/s (42%). Stier-Jarmer et al. [6] analyzed the hospital charts of geriatric inpatients of five different hospitals in Europe. Most participants had functional limitations with mobility activities at admission and discharge, such as changing basic body position (68 and 39%, respectively) or walking (79 and 61%, respectively).

These mobility limitations can lead to a reduced physical functioning and physical activity. There is strong evidence that many older hospital patients are physically inactive most of the time, spending only 1–2 h per day in a standing position or walking [7–13]. For example, Brown et al. [7] reported that geriatric inpatients spend on average 20 h in bed, 3 h in a sitting position and only 43 min per day standing or walking. The median daily step count of 740 steps (interquartile range: 89 to 1014) reported for a cohort of 239 acute geriatric inpatients with a mean age of 77 years [14] is significantly lower than the normative value of 2026 to 3011 for the corresponding age group [12]. Physical activity levels are particularly low when therapy was not available: in the late afternoon, during the evenings and on weekends [13].

Low mobility in older hospital patients is significantly associated with negative health outcomes such as falls [15, 16], re-hospitalization [17], incident disability [18, 19], institutionalization [20, 21] and mortality [2, 21]. Thus, improvements of mobility capacity in older hospital patients are a major goal in geriatric care.

Early physical rehabilitation programs, including physical exercise and physiotherapy, offered immediately after hospital admission, are interventions aimed to increase mobility capacity and physical activity. These exercise interventions are usually delivered as a component of a multidisciplinary intervention [22–24]. There is evidence from three systematic reviews that early physical rehabilitation interventions seem feasible and can be executed safely in acutely hospitalized older adults [22–24]. However, evidence of the effects of exercise on clinical outcomes in this population is inconclusive. A systematic Cochrane review by de Morton et al. published in 2007 [22, 25] reported a significant increase in the proportion of patients discharged to their home (as opposed to another healthcare institution), and a small but important reduction in acute hospital length of stay and total hospital costs for multidisciplinary interventions that included exercise compared with usual care. The effect of exercise alone on functional outcome measures was unclear and there was no effect on length of stay or discharge destination in the acute setting. More recent reviews by Kosse et al. (2013) [23] and Martinez-Velilla et al. (2016) [24] report a positive effect on physical functional outcomes in most of the included studies, but the amount and content of the physical component of the early physical rehabilitation programs was reported insufficiently in many studies.

In Germany, the Diagnosis Related Groups (DRG) allow to classify procedures for geriatric hospital patients in the hospital reimbursement system. A standard procedure is the so called “early rehabilitation in geriatric medicine” (“Geriatrische Frührehabilitative Komplexbehandlung”, GFK) [26]. A GFK procedure is applied for older acute hospital patients and includes, at a minimum standard, the following aspects: (1) Care is delivered by a multidisciplinary team, including medical doctors, nurses, physiotherapists, occupational therapists, speech and language therapists and other allied health professionals. (2) A geriatric assessment immediately after admission and at discharge. (3) An assessment of the patient’s social and environmental situation. (4) Multidisciplinary team meetings once a week. (5) Activating-therapeutic nursing care. (6) At least two of the following four treatments: Physiotherapy/physical therapy, occupational therapy, speech and language therapy, psychology/neuro-psychology.

There are three main procedure codes (OPS) that apply for the GFK according to the length of care: OPS 8–550.0 for 7–13 days with at least 10 treatment sessions; OPS 8–550.1 for 14–20 days with at least 10 treatment sessions; OPS 8–550.2 for ≥21 days with at least 10 treatment sessions [26]. The mean duration of sessions needs to be at least 30 min and no more than 10% of treatments may be group-based sessions. Usually, patients receive daily physiotherapy in combination with other treatments according to the individual rehabilitation needs. In 2010, 73% of geriatric hospital cases in Germany were classified as a GFK procedure, with 50% coded as OPS 8–550.1 [26]. Although the GFK system aims to offer early physical rehabilitation, it is unknown if the amount of treatments is sufficient to improve mobility capacity and physical functioning in older geriatric patients. Additional exercise interventions may be beneficial.

Some studies have investigated the effects of additional exercise interventions and reported inconclusive results [27–35]. While some studies [27–29, 35] reported no significant reduction on length of stay, Oestergaard et al. [32] found a significant mean difference of 2.4 days induced by the implementation of an additional chair based exercise program. Positive effects on functional outcomes, fall rates and mobility were reported in most studies [28, 31, 33, 34], but some authors reported no benefits of additional exercises on physical functioning [27, 32, 35], discharge destination (home), mortality, intensive care admissions, falls [29] or incident delirium [30]. However, these studies differ with respect to the character, amount and intensity of the additional intervention (e.g. chair based exercises [32], extra walking [31], individually tailored physiotherapy led exercises [29]), the amount of usual care exercises and the duration of the intervention.

In line with others [27–29, 33], McCullagh et al. [36] suggested that the published studies failed to prove a significant reduction in length of stay due to methodical flows, such as using inappropriate outcome measures, including participants with good baseline physical performance levels, and poor adherence to the additional exercise interventions. These authors [36] thus published a study protocol for a randomized controlled trial (RCT) on the effects of an augmented prescribed exercise program (APEP) for frail older medical patients in the acute setting in Ireland. First analyses from a sample of 190 participants show that the intervention reduced length of stay slightly, but did not reach statistical significance (Hazard Ratio: 1.09 (95% CI: 0.77–1.56), p = 0.6) [37]. Physical performance was significantly and meaningfully better in the intervention group at discharge, but this effect was lost at follow-up.

Usual geriatric care in Ireland differs from GFK procedures commonly applied in Germany. In the McCullagh study [36], routine physiotherapy will be delivered three times a week, which is only 60% of the daily physiotherapy sessions usually offered according to the GFK. The feasibility and effect of APEP on mobility and physical functioning of older acute medical patients with a relatively high amount of usual care exercises (such as GFK procedures applied in Germany) has not yet been examined and is best tested in a RCT.

Before performing a large RCT (phase III study), a pilot study needs to prove the safety of the intervention and the feasibility of the study protocol [38]. Thus, the aims of this phase II randomized controlled pilot study were (1) to assess the feasibility of an augmented prescribed exercise program (APEP) in older acute medical patients and (2) to measure the potential effects of APEP on mobility capacity in order to assess the feasibility of a large full-scale study.

## Methods

### Trial design

We conducted a single-center, prospective, parallel-group randomized controlled pilot and feasibility trial. Participants were allocated to the intervention or control group by 1:1 ratio.

The study was approved by the Ethical Review Board of the German Confederation for Physiotherapy (registration number: 2016–12) and all participants provided written informed consent. The study protocol has been registered a priori in the German Clinical Trial Register (DRKS00011262). Reporting followed the CONSORT 2010 statement for randomized pilot and feasibility trials [39], the Template for Intervention Description and Replication (TIDieR) checklist [40] and the Consensus on Exercise Reporting Template (CERT) [41]. The study design was informed by the methodical procedures described by McCullagh et al. [36].

The study was initially planned from 31th Oct 2016 to 16th Dec 2016 only (47 days). Recruitment rates were lower than expected (n = 13). Thus, we set up a second study phase from 31st Oct 2017 to 5th Dec 2017 (36 days) in which we included a further 22 participants.

### Participants

Participants were recruited from the two acute medical geriatric wards of the ‘Evangelisches Krankenhaus Castrop-Rauxel’, a general academic teaching hospital with eight specialized clinics in a metropolitan area in the western part of Germany.

We included male and female older acute medical patients with (1) a minimum age of 65 years. Further inclusion criteria were (2) planned acute-geriatric stay of at least two weeks in the study hospital, (3) care plan according to “early rehabilitation in geriatric medicine” (GFK) procedures, (4) walking ability (with or without walking aid; independent or with stand-by assistance), indicated by a Functional Ambulation Categories (FAC) score ≥ 3 [42]), and (5) limited mobility, indicated by a timed up and go test (TUG) score of > 9 s [43].

Exclusion criteria were (1) significant cognitive impairment, (1a) which exceeds a slight cognitive impairment (ICD-10-Code: F06.7), and/or (1b) is documented with a validated test of cognition (e.g. Mini Mental State Examination (MMSE) < 18 points [44]), and/or (1c) due to the diagnosis of dementia, (2) severe hearing impairment, (3) severe visual impairment, (4) German language barrier, (5) acute psychiatric condition (e.g. delirium), (6) initiated palliative care, (7) any medical restriction for physiotherapeutic interventions (e.g. physical training), (8) lack of understanding of simple orders, (9) if the baseline-assessment had not been completed within the first 5 days after hospital admission.

Study procedures were held constant between the two study phases except for the exclusion criterion #9. Intentionally, participants were excluded if the baseline assessment was not performed within five days after admission (five exclusions in phase A). However, prior to performing the baseline assessment, study participants needed a prescription for physiotherapy made out by the ward physician, who also checked exclusion criterion #7 (medical restriction for physical exercise interventions). For many participants, the medical approval was not given soon enough after admission. To avoid exclusion of eligible study participants solely based on delayed hospital admission procedures, exclusion criterion #9 was changed for the second study phase in the following way: “baseline-assessment has not been completed within the first five days after *initial physician’s prescription for physiotherapy”*.

In the two recruitment periods, every older patient admitted consecutively to a geriatric ward of the study hospital was screened for eligibility by research assistants. At first, a ward physiotherapist screened medical charts of all patients. Potentially eligible participants were then visited by undergraduate physiotherapy students who informed patients about the study and assessed eligibility criteria not assessable from the medical charts, such as FAC score. Patients had at least one day to consider and discuss study participation with relatives. Approval and prescription for usual care physiotherapy needed to be confirmed by the ward physician. Written informed consent was obtained before conducting the baseline assessment.

### Interventions

Both groups received usual care, which included medical care and early rehabilitation interventions according to GFK standards [26]. The GFK was provided by a multidisciplinary team, including medical doctors, nurses, physiotherapists, occupational therapists, speech and language therapists and other allied health professionals.

All participants routinely received a Comprehensive Geriatric Assessment, which covered at least the following five domains: mobility, disability/activities of daily living, cognition, emotions and social care. The characteristics of the GFK have been described in the introduction. In summary, all participants were offered at least 10, 20 or 30 therapy sessions within 7, 14 or 21 days, respectively. Usually, individual physiotherapy was offered daily from Monday to Friday, with additional individual occupational and speech and language therapy sessions and group exercise classes. All rehabilitation interventions were usually performed by graduated clinical staff, aimed to improve the patients’ independence, activities of daily living (ADL) functioning and quality of life. Physiotherapy sessions were delivered by the clinical ward physiotherapists, designed to improve mobility, lower limb strength and/or balance depending on participants’ functional status and goals. Routine physiotherapy treatments consisted of assessment, exercise, provision of aids and other rehabilitation treatments, such as pain-relieving treatments, if appropriate, and discharge planning. It was mainly focused on active interventions to improve a patient’s physical functioning.

#### Control group

Participants in the control group received usual care (GFK) as required throughout their hospital stay, without any further interventions. Study assessment outcome scores were not reported to control group participants or usual care providers.

#### Intervention group

In addition to usual care, participants in the intervention group received APEP, which consisted of additional individual physiotherapy sessions scheduled 4–5 times per week. APEP sessions aimed to improve solely functional mobility capacity, so that no treatments for other medical issues, such as back pain or shoulder and arm disabilities, were delivered. Each APEP intervention period started immediately after randomization and lasted until participant’s hospital discharge, or for a maximum of three weeks.

The APEP intervention was individually tailored based on the functional abilities and needs of each participant. The starting level was defined according to the results of the individual Comprehensive Geriatric Assessment and the individual test scores of the study baseline assessment. There was no pre-defined protocol or set of exercises which was used for a defined group of participants. Instead, the selection, intensity and progression of the APEP physiotherapy sessions were individually directed by the exercise providers.

All APEP physiotherapy sessions were performed individually, delivered and supervised by one of four different graduated physiotherapists with ≥2 months of working experience (KS, SE, MW, IS), but with ≥3 months of practical experience in geriatric rehabilitation (practical internships during bachelor studies). The exercise providers were well informed about the background of the study, the concept of the study and the evidence of additional exercises for older hospital patients.

Each session was scheduled for 20 to 30 min and followed the same structure: [1] Brief assessment of the participant’s recent condition, motivation and recovery level. [2] Warm up (3 min). [3] Main part (15–20 min). [4] Cool-down (2 min). [5] Brief post-test, including BORG assessment of session perceived exertion [45]. The warm up consisted of low-intensity chair-based exercises to stimulate the circulation or by walking to the ward therapy room. The main part included at least three strengthening exercises, followed by 2–3 balance-, endurance and/or walking exercises. The cool down was usually walking back to the participant’s ward room.

For each APEP session, the date, time, duration and (if appropriate) reasons for abortion were documented. The participant’s recent condition was evaluated by subjective reports and consultation of the participant’s hospital chart. The participant’s motivation for each additional physiotherapy session (APEP) was assessed with an 11-point Likert numeric rating scale (0 = not motivated at all; 10 = highly motivated). Physical recovery was also rated on an 11-point Likert numeric rating scale (0 = not recovered/very exhausted; 10 = highly recovered). Participant’s subjective rating of perceived exertion was assessed with the 15-grade BORG rating scale (6–20 points) [45].

The functional exercises (main part) aimed to increase the participants’ functional mobility, including transfer abilities, walking stability, static and dynamic balance, and walking endurance by focusing on lower limb strength, balance and coordination, and cardiorespiratory fitness. The exercises were taken from three different evidence-based intervention programs designed to increase physical functioning, balance and mobility of older people: The Weight-bearing Exercise Program (WEBB) [46], the Otago Home-based Exercise Program (OEP) [47] and the High-intensity Functional Exercise Program (HIFE) [48]. Typical exercises to improve lower limb strength were heel raises, partial squats, sit-to-stand, stepping forward and sideways up onto blocks and stair-walking. Dynamic and static balance was typically trained in a standing position with a reduced base of support, such as closed feet or (semi) tandem stance position. Breaks were offered as needed. The selection of individual exercises was based on the individual needs, abilities and preferences of each participants and left to the competence of each exercise provider (physiotherapist).

Exercise equipment was used according to individual exercise programs and feasibility for hospital exercise training with older people. Typical equipment were soft balls, tissues, balance pads, rods, mirrors, dumbbells (< 10 kg), resistance bands and handrails. For some exercises, everyday objects, such as a chair, a staircase or the hospital bed were used. APEP exercises were performed in the participant’s ward room, a therapy room on the hospital ward and/or on the ward floor or staircase (gait and stairclimbing exercises).

APEP physiotherapy sessions were scheduled 4–5 times a week for each participant over a period of three weeks. The intervention was abandoned if the participant was discharged earlier. APEP sessions were usually scheduled in the afternoon, after completion of the usual care rehabilitation interventions. Each session was scheduled for 20–30 min, and reasons for individual abortion were documented. The intensity of each session was individually tailored and guided by three parameters: (1) Participant’s subjective rating of perceived exertion assessed with the 15-grade BORG rating scale (6–20 points) [45]. (2) Participant’s subjective report of load, fatigue and well-being. (3) Participant’s outer impression, such as face color, heart rate, breathing or quality of movements. Strengthening exercises were performed with a moderate to high intensity, with 3 sets of 13–15 repetitions each (BORG 12–14 after each set). The aim was to train 15–20 min without adverse events, and to record perceived exertion using BORG at the end of each session.

Participants were encouraged to be active and to walk on the hospital ward as much as possible, according to the daily (current) perception and level of recovery. Patients were not instructed to exercise without supervision or in addition to the APEP intervention. However, if unsupervised exercises were prescribed as part of usual care, patients were encouraged to perform these exercises. No non-exercise components were provided.

The APEP intervention was delivered according to the principles described above. We did not alter these principles throughout the study.

Adherence to the APEP sessions was recorded as described above. Apart from explaining potential benefits of APEP, participants were not specifically motivated by physiotherapists to adhere to the additional APEP sessions. If a participant refused or aborted an APEP session, we recorded the individual reason. We also recorded any potential moderate or severe adverse events during exercise sessions, such as falls, cardio-respiratory complications, vertigo, injuries as well as any reports of muscle tenseness, strains or pain. Minor adverse events were not recorded since these events were considered common in usual care physiotherapy with acute older medical patients. Adverse events were classified as follows:

1. *minor*: The issue can be solved/treated by providing a short break. A consultation of the ward physician is not necessary. The assessment/exercise session is continued on the same day (e.g. brief vertigo of minor intensity).
2. *moderate*: The assessment/exercise session must be aborted. A consultation of the ward physician is not necessary. The assessment/exercise session is continued on another day (e.g. acute joint pain with remission on the following day).
3. *severe*: The assessment/exercise session must be aborted. A consultation of the ward physician is necessary. The assessment/exercise session may be continued on another day (e.g. fall without injury) or the participant is not able to continue the assessment/exercise session directly (e.g. fall with fracture).

Any severe adverse event was reported to the responsive usual care physiotherapists, the responsive ward physician and the responsive ward nurse.

### Outcomes

#### Feasibility outcomes

The feasibility of the study protocol for a potential main study was assessed along a set of criteria (Table 1) which were informed by the recommendations given by Thabane et al. [38].

**Table 1:** Feasibility outcomes of the study

Outcome	Description	Operationalization and unit
Feasibility of the processes and the study design that are key to the success of the main study		
Recruitment rate	Rate between older acute medical inpatients admitted during the study period and study participants	n participants/n inpatients [%]
Recruitment time	Time needed to recruit 30 participants	Time [weeks]
Refusal rate	Rate between inpatients declining to participate and eligible inpatients	n refusing inpatients/n eligible inpatients [%]
Retention rate	Rate between study drop-outs and study participants	n drop-outs/n of participants included [%]
Eligibility criteria	Is it obvious who meets and who does not meet the eligibility requirements?	n of unclear cases
	Are the eligibility criteria sufficient or too restrictive?	Sufficient if recruitment rate > 10%
Randomization	Comparability of randomized groups (blocks of 8 without stratification)	Primary clinical endpoint (de Morton Mobility Index): Assessment of statistical differences between intervention and control group at baseline (2-sided t-test)
Feasibility of the outcome measures		
Safety	Number of adverse events during study assessments	Total n
Duration	Length of time to fill out all the study forms	Time in minutes; number (rate) of assessment sessions completed within one single session
Acceptance	Number of refused outcome measures	Total n
Completeness	Number of missing items per outcome measure	Total n
Interpretability	floor or ceiling effects	≥15% of participants are not able to perform an outcome measure; ≥15% of participants score the lowest or highest score of a scale
Resource problems that can occur during the main study		
Equipment	Is the equipment readily available when and where it is needed?	Number of issues with assessment equipment, such as break downs, shortage of equipment
Center willingness and capacity	Do the clinical centers do what they committed to do?	Number of issues with workflows; number of capacity issues
Feasibility and acceptability of the APEP intervention and adherence to the exercise protocol		
Amount	Frequency	n of sessions per participant; mean n of sessions per weak
	Duration	Mean duration of sessions [min]
Compliance	Rate of scheduled and completed sessions	N sessions completed / n sessions scheduled (%)
Intensity	Participant’s subjective rating of perceived exertion assessed	Mean perceived exertion after each session [BORG scale score]
(Non)compliance or adherence rate	Rate between intervention group participants who perform at least 75% of planned APEP intervention sessions and all intervention group participants	n of intervention group participants who perform at least 75% of planned APEP intervention sessions/n intervention group participants [%]
Safety	Adverse events (moderate, or severe) caused not, probably caused, likely caused or surely caused by the intervention	Total n
Acceptability	Motivation	Mean motivation of participant’s to perform additional physiotherapy sessions (APEP) [numeric rating scale score; 0–10 points]
	Recovery	Mean participant’s recovery prior to additional physiotherapy sessions (APEP) [numeric rating scale score; 0–10 points]
Costs	Delivery of APEP intervention	Costs [€] for all actually planned and delivered APEP sessions

Abbreviations: *n* = number, *APEP* = augmented prescribed exercise program

To assess potential human and data management problems, we performed semi-structured interviews after phase A with the following groups involved in the study: study coordinators in the hospital, research staff performing the outcome measures, exercise providers. We aimed to assess, in open questions, the following potential issues: (1) Effort and challenges in the eyes of the participating hospital with managing the study. (2) Challenges of the study personnel in the hospital. (3) Challenges during organization and conduction of study assessments. (4) Challenges during organization and conduction of APEP intervention.

Assessments to estimate the potential treatment effect, the variance of the treatment effect and the sample size needed for the main study are described in detail further below.

To calculate the costs for the additional exercise interventions (APEP), we recorded the total number of actually planned APEP sessions and multiplied this number with the personnel costs in Euro for 30 min of physiotherapy treatment in the study hospital (23.16€ gross for 60 min = 11.58€ gross for 30 min).

#### Clinical outcomes

Outcomes to describe the study population at baseline were age, sex, cognition (MMSE), disability (Barthel Index), primary diagnosis, walking aid and multimorbidity. These data were taken from the participants’ hospital charts.

To estimate the potential treatment effect, we assessed a set of clinical outcomes relevant to older hospital patients. The primary clinical outcome was the change in mobility capacity from baseline to follow-up assessed with the de Morton Mobility Index (DEMMI). Secondary clinical outcomes were the changes in mobility, balance and ambulation, physical endurance, frailty and falls-efficacy (Table 2). A further secondary outcome was length of stay (in days) in the acute setting, measured from acute hospital admission to discharge, e.g. to a rehabilitation institution or back home.

**Table 2:** Outcome measures used in the study

Domain	Outcome measure	Scale range	Metric	ORO	PRO	Baseline	Follow-up
Mobility	de Morton Mobility Index	0–100 points	ratio	X		X	X
	Gait speed	ratio [m/s]	ratio	X		X	X
	Hierarchical Assessment of Balance and Mobility	0–26 points	ratio	X		X	X
	Timed up and go test	ratio [s]	ratio	X		X	X
Walking ability	Functional Ambulation Categories	0–5 points	ordinal	X		X	X
Physical endurance	6-min walk test	ratio [m]	ratio	X		X	X
Frailty	Frailty Index	0–1 points	ratio	X	X	X	X
Falls efficacy	Falls Efficacy Scale – International	16–64 points	ordinal		X	X	X
Multimorbidity	Number of comorbidities	ratio [n]	ratio		X	X	
Disability	Barthel Index	0–100 points	ordinal	X		X	
Cognition	Mini Mental State Examination	0–30 points	ordinal	X		X	
Length of stay	Length of stay	Ratio [days]	ratio				X

*ORO* = observer-rated outcome (performance-based), *PRO* = patient-reported outcome, *m* = meter, *s* = seconds, *n* = number

Clinical study outcomes were assessed at two times: Baseline assessment was completed within 5 days after hospital admission (first study phase) or 5 days after initial prescription for usual care physiotherapy (second study phase). Follow-up assessment was completed after a minimum of 14 days after hospital admission, and as close as possible to discharge or after a maximum of three weeks after baseline assessment. All data were collected by specifically trained undergraduate physiotherapy students blinded to group allocation except for the Barthel Index and MMSE, which were assessed by the occupational therapy and nursing staff of the hospital as part of routine care immediately after admission and prior to discharge. Each study assessor received a training (theoretically and practically) of 6–8 h on the study outcome measures.

All study assessments were performed in the patient’s hospital room and on the ward. Breaks were offered between outcome measures. A hand-held digital stopwatch was used for all temporal outcomes. Walking distances were recorded with a digital measuring wheel. Patients were scored at their highest level of safe function, using their usual walking aid. The same device was used for all assessments in a single session. Descriptions of several study assessments used here are based on previous publications [49–51].

The *de Morton Mobility Index (DEMMI)* is a bedside assessment, consisting of 15 hierarchical mobility items dealing with bed and chair mobility, ambulation, static and dynamic balance [52]. The DEMMI is scored using a Rasch analysed ratio scale (0 to 100 points). Higher scores indicate better mobility. The minimal important change for older acute medical patients is 10 DEMMI points [52]. A German language DEMMI version was used in this study [51, 53, 54].

The *Hierarchical Assessment of Balance and Mobility (HABAM)* is a clinical bedside mobility assessment providing interval level data. Higher scores indicate higher mobility functions (0 to 26 points) [55, 56]. A validated German language version was used [51, 57].

Habitual *gait speed* in m/s was assessed over a distance of 4.6 m (15 ft). Timing was started when the participant began walking. The mean time of two trials was used for analysis [58, 59].

The *Timed Up and Go test (TUG)* is a performance-based test that assesses basic mobility functions. The patient is asked to stand up from a chair, walk 3 m, turn around, walk back and return to the chair [43]. In the present study, the TUG was performed as originally described [43], but a cone had to be encircled and participants chose the turning side. A familiarization trial was followed by one counted trial. Shorter times indicate higher mobility.

The *Functional Ambulation Categories (FAC)* distinguishes 6 levels of walking ability subjected to the amount of assistance required over a walking distance of 10 m [42, 60]. Lower scores, where physical assistance is needed, indicate poorer mobility than higher scores, where the patient is able to ambulate independently.

The *6-min walk test* was used to quantify walking endurance and functional-exercise capacity [61]. Subjects were asked to walk as far as possible within six minutes on the hospital corridor. Only one trial was performed to avoid fatigue effects. Distance was scored in meters.

The *Frailty Index* is a measure of frailty according to the model of deficit accumulation, based on a set of health-related variables [62]. In this study, a 40-item Frailty Index was calculated based on the variables proposed by Searle et al. [63]. The score of the Frailty Index is the ratio of health deficits present to the total number of health-related variables. Peak flow, shoulder strengths, grip strength and gait speed (habitual and fast) were rated based on actual physical performance. All other items were patient-reported. Shoulder strength (one trial) of the dominant arm was measured with a hand held digital dynamometer (Biometrics MicroFET2). For grip strength, the highest score of 3 trials in the dominant hand was the final score. A JAMAR dynamometer (Patterson Medical, Model 5030 J1) was used following the assessment protocol proposed by Roberts et al. [64]. Gait speed was assessed as described above.

The *Falls Efficacy Scale-International (FES-I)* is a patient-reported outcome measure to assess falls efficacy [65, 66]. The person is asked to rate his or her concerns regarding falling while performing several ADL situations on a 4-point Likert-scale (“not at all concerned” to “very concerned”; 16 to 64 points). Higher values represent more concerns in fall-prone situations.

The *Barthel Index (BI)* [67] (0–100 points) is a performance-based measure of functioning and independence in the ADLs. Higher scores indicate better functioning. In this study, the BI was applied by the nursing staff as part of routine care within the first 7 days after admission.

The *Mini Mental State Examination Test (MMSE)* is an 11-item assessment of cognitive function (0 to 30 points) [68].

*Multimorbidity* was defined by ≥2 of the following 8 self-reported chronic disease: high blood pressure, history of heart attack, chronic heart failure, history of stroke, history of cancer, diabetes mellitus, osteoarthritis/arthritis, and chronic lung disease. If a condition was suspect or diagnosis was unclear, it was not counted for comorbidity.

As described in Table 1, the feasibility of obtaining the outcome measures was assessed with respect to safety, duration, acceptance, completeness and interpretability. For the main study, we decided a priori to leave out an outcome measure if one of the following criteria was matched: [1] occurrence of at least one serious adverse event during assessment. [2] Outcome measure refused by ≥10% of participants. [3] At least 10% of missing items in ≥10% of assessments. [4] A floor or ceiling effect of ≥15% [69].

The DEMMI was chosen as the primary outcome of mobility based on its excellent content and construct validity, reliability and responsiveness in older acute medical patients [50, 52, 70]. In a pilot study on enhancing physical activity in older hospital patients, Said et al. [33] determined the most appropriate measure of mobility by comparing the DEMMI with the Elderly Mobility Scale and the TUG. In contrast to the latter scales, the DEMMI showed no floor or ceiling effects and was thus described as the strongest measure of mobility. We used some other measures of mobility (HABAM, TUG, gait speed) to assess which instrument is most appropriate for measuring mobility in the main study.

### Sample size

This pilot study is used to generate information for sample size calculation for the main study. According to Whitehead et al. [71] and Thabane et al. [38] formal power considerations are usually not necessary for a pilot study. However, the target sample size for this pilot study is 30 participants based on the sample size recommendations for pilot studies [71]. There will be no interim analyses or stopping guidelines.

### Randomization, allocation concealment mechanism and implementation

Participants were randomly allocated to the intervention (APEP) or control (usual care) group. A computer-generated randomization list with blocks of eight was created and used for group allocation. This list was stored and managed by an independent investigator in the clinic (randomization manager). The randomization list was not accessible to the assessors and the allocation sequences were concealed until baseline assessment and informed consent was completed.

Enrollment of participants was performed by undergraduate physiotherapy students, supported by the study coordinator in the clinic (KS). These people assessed eligibility, informed patients about the study, obtained informed consent, performed the baseline assessment and reported study participants ready for randomization to the randomization manager. After randomization, group allocation was reported to the exercise providers, who also informed participants on group allocation.

### Blinding

The study assessors were blinded towards group allocation at all times. Blinding was not possible for participants, exercise providers and the study coordinator in the clinic. Participants were intensively urged not to mention their group allocation during follow-up assessment to avoid un-blinding of study assessors. The data analysts (TB, CT) were blinded by coding the group allocation in the data file.

### Analysis

Data were managed by the principal investigators (TB, CT). For the statistical analyses, SPSS statistical software was used (Version 25.0, SPSS Inc., Chicago, IL, USA).

Feasibility outcomes were reported descriptively and narratively. For the primary and secondary clinical outcomes, medians (range) were reported for ordinal data, mean (standard deviation) for continuous data and raw count (number, %) for nominal data. Confidence intervals (95% CI) are reported if appropriate.

Length of stay and relevant baseline differences between the control and intervention group were compared by two-tailed t-test (length of stay, age, MMSE, Barthel Index, DEMMI), Mann–Whitney U test (FAC) or chi-square test (sex, multimorbidity).

All outcome measures were summarized and 95% confidence intervals constructed for the difference in outcomes between control and intervention groups. Change scores were calculated by subtracting follow-up scores from baseline scores. Analysis of covariance (ANCOVA) was used for mean differences in continuous outcomes, with adjustment for baseline values to control for baseline imbalances because of regression to the mean [72]. Both adjusted and unadjusted values were tested to detect which had the smaller variance. Mann–Whitney U test was used to compare median change scores in ordinal outcomes (FES-I and FAC). All efficacy analyses were two-tailed.

For all continuous outcomes, Cohen’s effect size (ES) *d* was calculated [73] as mean change over time for the intervention group minus the mean change over time for the control group, divided by the pooled SD across the two measurement occasions. For all ordinal outcomes, the ES *r* was calculated, defined as $r=\frac{\mathrm{Z}}{\sqrt{\mathrm{N}}}$, where Z is derived from the Mann-Whitney U test statistics, and N the number of observations included [73]. Thresholds for ES according to Cohen were: 0.20 = small, 0.50 = medium, 0.80 = large [74].

All analyses were performed according to the intention-to-treat principle where the ‘last observation carried forward’ method was used to address missing follow-up data. The level of statistical significance was set to p < 0.05. No alpha-correction was applied due to the exploratory character of this pilot trial.

We hypothesized that intervention group participants with an APEP adherence of ≥75% would show larger improvements in DEMMI scores than participants with a low adherence. Thus, we performed a subgroup analysis by comparing the mean DEMMI change scores of these two groups (independent t-test).

The sample size calculation for the main superiority study is based on Cohen’s *d* ES of the primary continuous outcome (mobility capacity assessed with the DEMMI) found in this pilot study, 80% power and two-sided 5% significance [71]. The software G*Power (Version 3.1.9.2 [75]) was used to calculate the sample size.

## Results

### Flow of participants through the trial

**Fig. 1:**
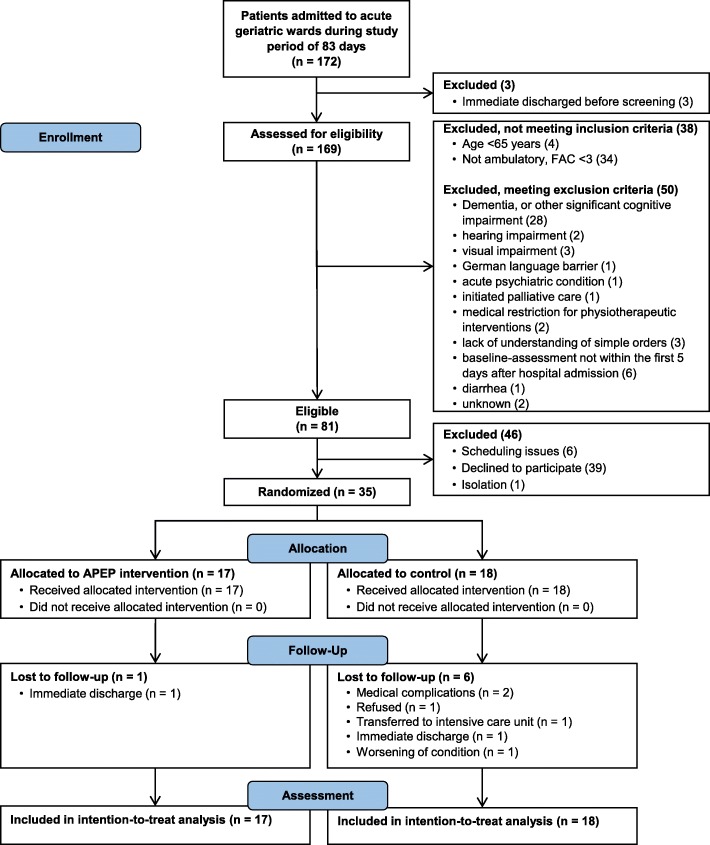
Participants’ flow through the trial

The flow of the participants throughout the study is illustrated in Fig. 1. A total number of 35 older acute medical patients (74% female) with an average age of 80.9 ± 7.7 years were included (Table 3).

**Table 3:** Demographic and clinical characteristics of the study participants at baseline

Characteristic	Intervention group (n = 17)	Control group (n = 18)
Age, years	78.6 ± 7.5	83.1 ± 7.4
Sex, female, n (%)	13 (76%)	13 (72%)
Mini-Mental State Examination, 0–30 points	25.7 ± 3.0	26.4 ± 2.8
Barthel Index, 0–100 points	62.9 ± 13.9	58.3 ± 17.2
Comorbidities, median (IQR)	2 (0–4)	2.5 (2–3.25)
Multimorbidity (≥2 comorbidities), n (%)	10 (59%)	15 (83%)
Time admission to baseline, days	2.4 ± 1.4	2.3 ± 1.3
Time baseline to follow-up, days	12.4 ± 2.6^a^	12.5 ± 2.1^b^
Time follow-up to discharge, days	3.7 ± 2.8	3.8 ± 1.7
Primary diagnosis according to ICD-10		
IV Endocrine, nutritional and metabolic diseases, n (%)	0	2 (11%)
VI Diseases of the nervous system, n (%)	3 (18%)	1 (6%)
IX Circulatory, n (%)	3 (18%)	3 (17%)
X Respiratory, n (%)	0	3 (17%)
XI Digestive system, n (%)	1 (6%)	0
XIII Musculoskeletal, n (%)	7 (41%)	5 (28%)
XVIII Symptoms, signs and abnormal clinical and laboratory findings, n (%)	2 (12%)	4 (22%)
XIX Injury, poisoning and certain other consequences of external causes, n (%)	1 (6%)	0
Functional Ambulation Category, median (IQR)	4 (3–4)	4 (3–4)
3, n (%)	5 (29%)	7 (39%)
4, n (%)	12 (71%)	10 (56%)
5, n (%)	0	1 (6%)
Walking aid		
None, n (%)	2 (12%)	4 (22%)
Wheeled-walker/rollator, n (%)	14 (82%)	13 (72%)
Cane/single crutch, n (%)	1 (6%)	1 (6%)

Abbreviations: *IQR* = interquartile range, *ICD-10* = 10th revision of the International Statistical Classification of Diseases and Related Health Problems

Data are mean ± standard deviation unless stated otherwise

^a^ n = 16 ^b^ n = 12

All intervention group participants received the allocated intervention. However, one participant (code #119) did not participate in any APEP intervention: the first two scheduled sessions were canceled due to reports of severe pain and aggressive behavior of the participant (delirium), respectively. No further APEP sessions were scheduled for this participant, who withdrew from the trial and who also missed the follow-up assessment due to immediate discharge within 2 weeks after hospital admission.

All participants in the control group received the allocated control intervention (usual care). The reasons for the 6 missing follow-up assessments are given in Fig. 1. The medical complications in 2 participants were: (1) One participant developed a pressure ulcer on the lower limbs. (2) One participant with severe chronic obstructive pulmonary disease (COPD, GOLD IV grade) was not able to perform any performance-based outcome measures due to a worsening physical condition. However, this latter participant performed the FES-I and all self-reported items of the Frailty Index (35 items), which were both included in the follow-up analyses. The participant’s missing data of physical outcome measures were imputed as described.

### Baseline data

The 17 APEP intervention participants and 18 control participants did not differ significantly in baseline demographic characteristics and baseline functional ambulation (Table 3). The imbalance between the number of participants with multimorbidity in the control group compared to the intervention group (15 versus 10) was not statistically significant (*p* = 0.11).

### Feasibility outcomes

#### Feasibility of processes

The recruitment rate was 20.3% (35/172). We needed a total of 83 days (12 weeks) to recruit 35 participants (2.37 days per participant). The refusal rate was 48.1% (39/81). The retention rate was 80%, since we lost 7 of 35 (20%) participants to follow-up.

There were no reports of unclear cases concerning the eligibility criteria. However, we altered the exclusion criterion #9 as described above. In phase A, we excluded 5 potential participants due to late baseline assessment. In phase B, all baseline assessments could be performed within 5 days after the initial physiotherapy prescription (median time between admission and prescription for physiotherapy: 0 (IQR: 0–1) days, n = 22).

The difference of 3.5 points (95% CI: − 6.0 to 12.9) in DEMMI baseline scores between both groups was not statistically significant.

#### Feasibility of the outcome measures

In 7 participants, no follow-up assessment was performed (Fig. 1). All remaining 63 study assessments were performed without any adverse events. Some participants, however, perceived the duration and character of the study assessments as too long and exhausting. We did not record the exact duration of each assessment session, but it was reported by the research personnel that a session took approximately 45–60 min, depending on the level of physical functioning and compliance of each participant. If participants reported fatigue, breaks were offered. We were able to complete each study assessment within one day. One participant refused the whole follow-up assessment and one participant could not perform the performance-based measures at follow-up (2.8%). All other outcome measures were completed as planned.

**Fig. 2:**
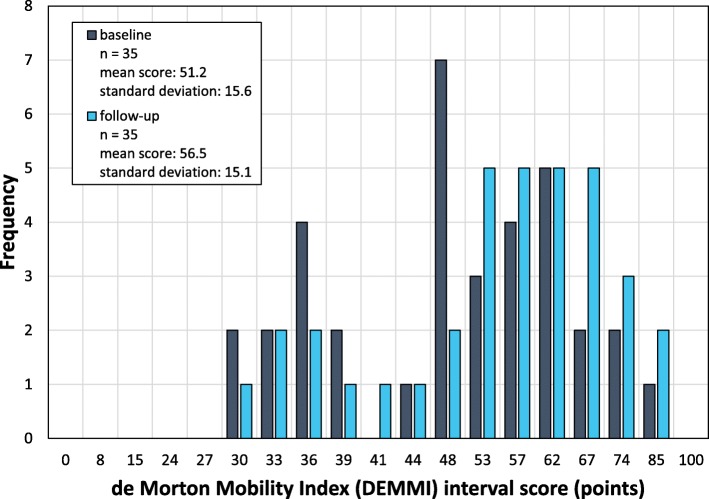
Distribution of de Morton Mobility Index scores

DEMMI score range was from 30 to 85 points at both time points (Fig. 2), following a normal distribution at baseline (p = 0.26) and follow-up (p = 0.38). There were no floor or ceiling effects.

DEMMI, HABAM, gait speed, TUG, FAC, 6-min walk test and FES-I data was available at baseline for all 35 participants (100%), and 27 of 28 (96%) participants performed these measures at follow-up. There were no missing items for any outcome measure except for the Frailty Index. At baseline, each Frailty Index was completed with 40 items. At follow-up (n = 28), the MMSE-item was missing in 10 participants. One participant did not conduct the performance-based items and one participant had 10 missing items on self-rated health, social activities and mood, so that those Frailty Indices were calculated with 35 and 30 items, respectively.

There were no floor or ceiling effects for any outcome measure at any time point (figure in Additional file 1).

#### Resource problems

We used two complete sets of assessment equipment, which were stored in the central physiotherapy office of the hospital. No significant issues were reported.

#### Feasibility and acceptability of the APEP intervention and adherence to exercise protocol

Sixteen (94%) of the intervention group participants participated in at least one APEP session. One participant (#119) did not receive any of two scheduled APEP sessions due to reports of pain and aggressive behavior, respectively. This participant was later withdrawn from the study due to immediate discharge.

**Fig. 3:**
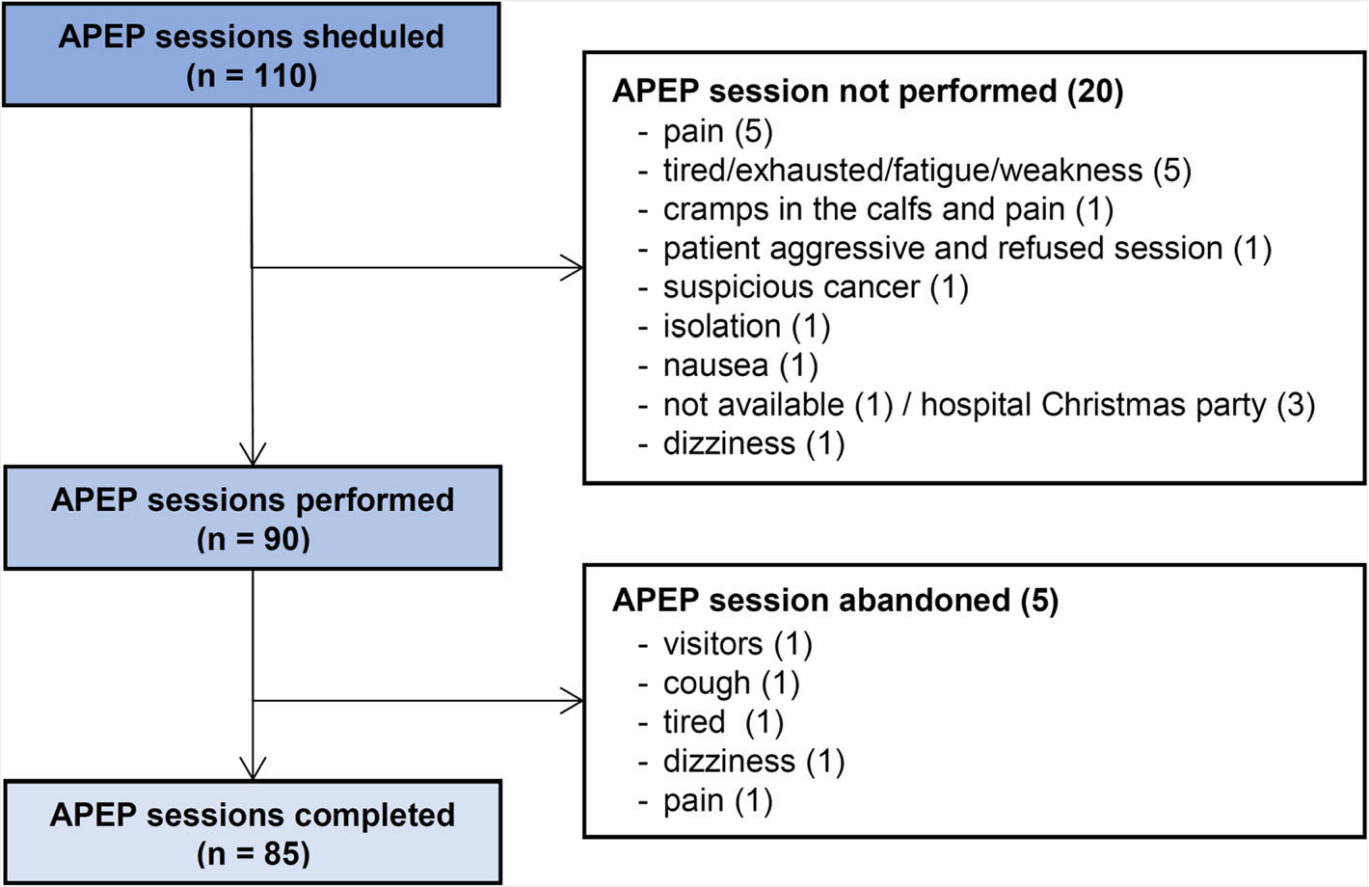
Overview of augmented prescribed exercise program (APEP) sessions with reasons for non-adherence and for abandonment

In total, 110 APEP sessions were scheduled for the 17 intervention group participants. Ninety APEP sessions (82%) were performed as scheduled. Reasons for non-adherence are given in Fig. 3 (canceled APEP sessions).

**Fig. 4:**
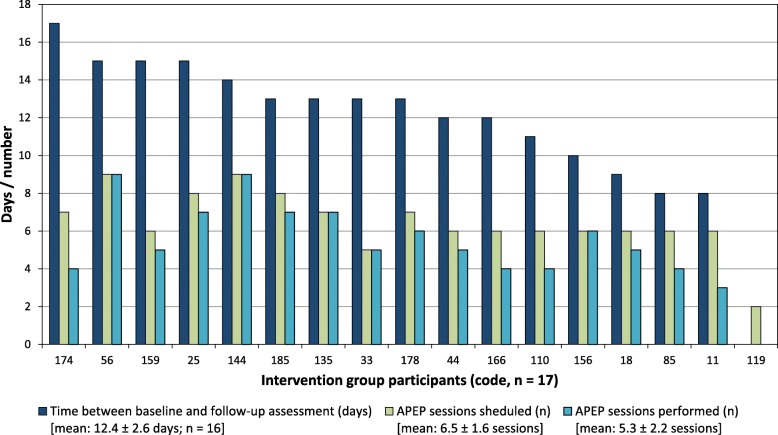
Number of scheduled and performed augmented prescribed exercise program (APEP) sessions per participant according to the duration of each participant’s individual intervention period. Participant #119 withdrew from the study without performing any APEP session or the follow-up assessment

Figure 4 illustrates the number of scheduled and performed APEP sessions per participant according to the duration of the individual intervention period. The mean time between baseline and follow-up assessment was 12.4 ± 2.6 (range: 8–17) days. The mean number of scheduled and performed APEP sessions was 6.5 ± 1.6 (range: 2–9) and 5.3 ± 2.2 (range: 0–9) sessions, respectively. Participants performed a mean of 3.0 ± 1.1 (range: 0–4.5) APEP sessions per week (7 days).

Of the 4–5 APEP sessions per week planned initially (=0.64 sessions/day) for each participant, 6.5 sessions were actually scheduled in 12.4 days (= 0.52 sessions/day), mainly because of time constraints induced by other usual care procedures and leisure group activities such as singing offered at the hospital.

The mean adherence rate was 78 ± 26% (range: 0–100) of scheduled APEP sessions. An adherence rate of ≥75% was observed in 11 participants (65%). This “high adherence subgroup” performed a mean of 6.5 ± 1.5 (range: 5–9) APEP sessions, whereas the “low adherence subgroup” (n = 6) participated in a mean of 3.1 ± 1.6 (range: 0–4) sessions.

The mean duration of the 90 APEP sessions was 23.2 ± 4.0 (range: 14–30) minutes. However, 5 APEP sessions were abandoned before completion due to visitors, cough, tiredness, dizziness or pain, respectively (Fig. 3). No such (moderate adverse) event let to a withdrawal from the study intervention in general, and there were no adverse events reported that were considered to be related to the APEP intervention. The mean duration of the abandoned APEP sessions was 15.2 ± 1.1 (14–17) minutes. The duration of all APEP sessions is illustrated in the histogram in Additional file 2.

The total amount of additional physiotherapy (APEP) was 2090 min (34.8 h) for 17 participants, which is 123 min on average per participant.

The level of motivation and recovery (subjective fitness to exercise) prior to each APEP session, the perceived physical exertion and the duration of the APEP sessions are given in Table 4.

**Table 4:** Motivation, recovery, perceived physical exertion and duration of the APEP sessions

	n sessions	Median
Motivation prior to each APEP session (0–10 points)	89	8 (IQR: 5.8–8), range: 0–10
Recovery prior to each APEP session (0–10 points)	89	6 (IQR: 5–8), range: 0–10
BORG scale (6–20 points)	83	14 (IQR: 12–15), range: 8–20
Duration of APEP sessions	90	22.5 (IQR: 20–25), range: 14–30

Abbreviations: *n* = number, *IQR* = interquartile range, *APEP* = augmented prescribed exercise program

The total costs for 110 scheduled APEP sessions were 1273.80€.

### Outcomes and estimations and ancillary analyses

Baseline, follow-up and change scores for the primary and secondary outcomes are given in Table 5. There were no statistically significant differences in mean change scores in any outcome measure. Participants in the intervention group tended to improve their mobility by 7.8 DEMMI points (16% of mean baseline value), while control group participants tended to improve by 2.8 points (5% of baseline).

**Table 5:** Unadjusted and adjusted mean between-group differences in the primary and secondary outcomes

Outcome measure	Intervention group, n = 17			Control group, n = 18			Unadjusted between-group differences		Adjusted between-group differences*		ES
	Baseline	Follow-up	Change	Baseline	Follow-up	Change	mean (95% CI)	p	mean (95% CI)	p	
DEMMI, mean (SD)	49.4 (16.0)	57.2 (17.0)	7.8 (12.7)	52.9 (11.1)	55.7 (11.3)	2.8 (9.1)	5.0 (−2.5 to 12.6)	0.18	4.1 (0.4 to 7.8)	0.26	0.45
HABAM, mean (SD)	19.1 (4.7)	20.3 (4.9)	1.2 (3.5)	19.9 (4.4)	20.9 (4.0)	1.0 (2.1)	0.2 (−1.8 to 2.2)	0.86	0 (−0.9 to 0.9)	0.99	0.07
TUG, mean (SD)	28.6 (13.2)	22.8 (12.2)	−5.8 (6.6)	24.9 (11.1)	22.4 (9.5)	−2.5 (5.9)	3.3 (−1.0 to 7.6)	0.13	2.5 (0.4 to 4.6)	0.21	0.53
Gait speed, mean (SD)	0.53 (0.17)	0.65 (0.20)	0.12 (0.20)	0.60 (0.19)	0.64 (0.28)	0.04 (0.15)	0.07 (−0.05 to 0.19)	0.22	0.07 (0.01 to 0.13)	0.25	0.45
6-min walk test, mean (SD)	154.5 (59.6)	194.9 (85.8)	40.4 (80.9)	167.7 (79.4)	170.8 (79.9)	3.1 (37.7)	37.4 (−5.6 to 80.4)	0.09	34.7 (13.7 to 55.7)	0.11	0.60
Frailty Index, mean (SD)	0.46 (0.20)	0.40 (0.19)	−0.05 (0.12)	0.46 (0.14)	0.41 (0.15)	−0.04 (0.08)	0.01 (−0.06 to 0.08)	0.72	0.01 (−0.02 to 0.05)	0.72	0.01
FES-I, median (IQR)	31 (22–57)	30 (22–52)	0 (−7–5)	31 (26–45)	31 (25–46)	0 (−4–4)	2.4 (− 5.5 to 10.3)	0.92	#	#	0.01
FAC, median (IQR)	4 (3–4)	4 (4–4)	0 (0–0)	4 (3–4)	4 (3–4)	0 (0–0)	0 (− 0.4 to 0.4)	0.81	#	#	0.04

Abbreviations: *n* = number, *ES* = effect size, *CI* = confidence interval, *IQR* = interquartile range, *DEMMI* = de Morton Mobility Index, *HABAM* = Hierarchical Assessment of Balance and Mobility, *TUG* = timed up and go test, *FES-I* = Falls Efficacy Scale – International, *FAC* = Functional Ambulation Categories

*Adjusted for baseline values of the respective outcome measure

The high adherence subgroup improved their mobility by 10.8 ± 12.1 DEMMI points, and the low adherence subgroup improved by 2.3 ± 12.9 points (*p* = 0.10).

The length of stay of intervention and control group participants was 18.4 ± 2.3 days and 17.8 ± 4.2 days, respectively (*p* = 0.62).

### Sample size estimation

Based on an ES of 0.45 for the DEMMI, assuming equally sized groups and a one-tailed significance threshold alpha of 5%, a total sample size of 124 participants would be required to detect a difference of 4 DEMMI points with a power of 80% in the main study. The recruitment of 124 participant in the same study hospital would take 294 days.

## Discussion

This randomized controlled pilot study provides important process, resource, management and scientific data, including a preliminary estimate of effectiveness, to guide the design of a future Phase III main study.

### Feasibility of process

Although only 47% (81/172) of participants were eligible, 20% of those patients approached during the study period were successfully recruited. These figures are comparable to other studies on additional exercises for older hospital patients [33, 34], e.g. Tibaek et al. [34] were able to recruit 27% of all potential participants (58% eligible).

The refusal rate of 48% seems comparably high [76] and should be reduced in the main study. We assume that the early study invitation, which was usually delivered on the admission day or within the first two days after admission, was the main reason for refusal in most patients. Anecdotal reports indicate that many patients were unaware of the acute care hospital procedures and they were afraid that additional exercises could lead to overextension. In the main study, the recruitment process should start not after potentially eligible patients have experienced 2 to 3 days of routine hospital care to better estimate the amount of rehabilitation services. The study information sheet may be reworked, e.g. with the involvement of former study participants, to better inform potential participants.

### Feasibility of outcome measure

The high retention rate (80%) and the high rates of completion of outcome measure assessments provide reassurance that the selected outcome measures were appropriate, safe and broadly acceptable to participants. Since some assessment sessions took up to 60 min, and this was reported to be too long and exhausting for some participants, one might consider to split the assessment session into two parts, performed on one or two separate days. Another option would be to adapt or reduce the total number of outcome measures. First, the 6-min walk test could be replaced by another valid but shorter test to measure physical endurance, e.g. the 2-min walk test [77]. Second, at least one measure of mobility could be left out, e.g. TUG and/or HABAM and/or gait speed, since the DEMMI seems to be an appropriate, feasible and highly valid instrument to measure mobility capacity in this population [33, 51, 70]. Third, since routine MMSE discharge data was missing for 10 of 28 participants, the study personnel should carefully screen follow-up data on cognition and, if required, perform the MMSE with the participants.

### Resources

The type of intervention and assessment in the current study does not need a lot of resources. The 2 complete sets of assessment equipment were sufficient and enabled parallel assessments. For a future main study in a bigger hospital or in 2 or more hospitals, the sets of assessment equipment should increase proportional to the number of participants per time.

### Feasibility and acceptability of the APEP intervention

Since we observed that the “high adherence group” showed higher improvements in mobility than the “low-adherence group”, a central aim of a future main study is to increase the number of performed APEP sessions per week (e.g. from 3 to 4) by increasing the number of offered APEP sessions and by increasing the adherence rate.

We initially planned to offer 4 to 5 additional physiotherapy sessions per participant per week. This was not possible within usual care procedures of the hospital, since participants received daily physiotherapy and regular group-based exercises, occupational therapy and other rehabilitation and medical treatments within GFK usual care procedures, as well as further group activities such as singing. We were able to offer a mean of 3.6 APEP sessions per week per participant (7 × 0.52 sessions/day). In order to offer further sessions in the context of GFK in Germany, it would be necessary to optimize the therapy planning procedures in the hospital, or to offer APEP sessions on weekend days [78].

The APEP adherence rate of 80% seems acceptable with respect to the largely frail and acutely ill study sample, also when considering adherence rates reported by others [33, 79]. Among the reasons for non-adherence or session abandonment (Fig. 3), no issues seem overrepresented or unusual for older acute medical patients. Twelve sessions (11%) were not performed or abandoned due to pain or tiredness/exhaustion/fatigue. Furthermore, the median self-reported recovery level prior to each APEP session was 6 of 10 points (Table 4), which may indicate insufficient recovery levels in some participants. Pain, sleep disturbances and fatigue are very common conditions in older people, challenging to manage and often inadequately treated [80–85]. In a future main study (as has been the case in the current study), intensive and adequate management of pain, sleep disturbances and fatigue within usual care procedures should enable affected study participants to participate in (additional) rehabilitation treatments, such as physiotherapy. APEP sessions should always be scheduled with appropriate time intervals prior and following usual (physical) care interventions to allow for sufficient recovery.

New evidence on how to increase the participation and adherence rate of the APEP intervention has recently been reported by O’Hare et al. [86], who explored how frail older inpatients perceived the effects of the APEP intervention. The authors concluded that “education tailored to the participants, and setting restorative goals, may improve outcome expectations and future intention to exercise” [86]. Especially the participants’ perceived relationship with the interventionist seems highly influential. It affects crucial aspects such as participation rate, perceived value of the APEP and outcome expectations [86]. We did not address and evaluate these aspects in our study, but it should be considered in future studies.

The present pilot study provides evidence that additional physiotherapy sessions are safe and feasible in older acute medical patients. These results are not surprising since the additional exercises included standard physiotherapy modalities and exercises from well-established training programs designed for older people. Moreover, these results are in line with previous studies which reported good feasibility of additional exercises in older hospital patients [27–35].

The aim was to deliver a substantial additional physical load to the intervention group participants with a duration of 20–30 min. The duration of the performed APEP sessions (mean 23.2 min) was sufficient. The median BORG scale score of 14 points (IQR: 12–15) indicates a moderate to high perceived exertion of the participants after the APEP sessions. While a potentially insufficient exercise intensity may compromise outcomes in this patient population [28, 29, 36], our results indicate that it may be difficult, and even unfeasible, to load the study participants in a way that the perceived exertion of BORG exceeds 15 at the end of each session.

### Outcomes and preliminary estimates of effectiveness

The present pilot study is not sufficiently powered to detect a statistically significant mean group difference in the primary outcome (mobility). To detect a difference in the pre-post change of the DEMMI of 4 points between the intervention and the control group, 124 participants would be needed (ES = 0.45, 5% alpha value, 80% power). It is debatable whether a mean difference of 4 DEMMI points would justify a larger main study, since this value is lower than the minimal important change of 10 DEMMI points reported for this population [52]. Ancillary analyses show a mean improvement of 10.8 DEMMI points in the “high-adherence group” with 5 out of 11 participants (45%) improving by 10 points or more, indicating that APEP may help older acute medical patients to improve their mobility capacity to a clinically relevant amount. In contrast, of the participants in the “low-adherence group” and in the control group, which showed a mean improvement of 2.3 and 2.8 DEMMI points, respectively, only 2 out of 6 participants (33%) and 5 out of 18 participants (28%) improved by 10 points or more. The adjusted between-group differences in the secondary outcomes (gait speed: 0.07 m/s; 6-min walk test: 34.7 m) did not reach the threshold of clinical significance [87, 88]. For the TUG (2.5 seconds) and the Frailty Index (0.01 points), no minimal important change values for older people have been published yet. Overall, a future main phase III study seems justified if feasible and convincing strategies to increase the APEP adherence rate are followed.

In a randomized clinical trial, Martínez-Velilla et al. [89] have assessed the effects of a multicomponent exercise intervention on the functional status in a sample of 370 acutely hospitalized older patients in a tertiary public hospital in Spain. The control group received usual care, including physical rehabilitation when needed. The in-hospital intervention included individualized moderate-intensity resistance, balance, and walking exercises (2 daily sessions). The authors reported that usual care was “offered to the patient by the geriatricians of our department and consists of standard physiotherapy focused on walking exercises for restoring the functionality conditioned by potentially reversible abnormalities. A formal exercise prescription was not provided at study entry” [89].

The study sample was about 10 times larger and slightly older than our sample (mean age: 87 years). Their median LOS (8 days) and median intervention duration (5 days) was shorter, and their usual care and intervention dose differ substantially from ours. This might explain why Martínez-Velilla et al. [89] reported significant benefits over usual care for physical functioning and disability, while we did not. Said et al. [35] published a multi-center, parallel-group, randomized controlled trial to assess if additional supervised physical activity leads to better mobility outcomes at discharge among 198 older people receiving inpatient rehabilitation. The experimental group received a median of 20 additional minutes of upright activities per day for a median of 16.5 days. Gait speed and DEMMI scores did not differ significantly between groups at discharge. These findings indicate that additional physical activity sessions did not lead to better mobility outcomes at discharge. The control intervention (individualized, multidisciplinary geriatric rehabilitation offered in Australian hospitals) seems comparable to the dose of usual care in the present study. This might indicate a limited benefit of additional exercise when there is already a high exercise dose and high quality of usual care. However, the study by Said et al. [35] included sub-acute rehabilitation patients, which differ from our acute medical population with respect to the functional status.

### Study limitations

We planned to deliver additional exercises 4 to 5 times per week. As some participants did not achieve the desired amount (‘dosage’) of the intervention, the potential effectiveness of the intervention may have been diluted. Moreover, the exact ‘dosage’ of physiotherapy or exercise interventions of the study participants is unclear, since we did not monitor the number, duration, content and intensity of usual care physiotherapy and other (physical) rehabilitation interventions. We assumed that the daily physiotherapy within the usual care GFK procedures were the same in both groups. While unlikely, the real dosage of “usual care physical exercises” may have differed between both groups, which would have introduced a bias.

We did not examine the effect of the intervention on other secondary outcomes, which have been used in comparable studies [33, 36, 90], such as discharge destination, readmission rate, disability, mortality, quality of life or physical activity. A core outcome set for effectiveness trials in older acute medical patients, which is not yet available, would be very useful to compare and pool the results of future studies on additional exercises in older hospital patients. Furthermore, we did not examine the longer-term impact of the APEP intervention on mobility, or any other secondary outcome. The inclusion of further outcome measures or potential follow-up measures in a future trial must be balanced with the burden on the study participants. We abstained from performing one or more follow-up measures, e.g. 3 months post discharge, due to the lack of resources for this pilot study.

Future studies should examine the cost-effectiveness of the APEP intervention, to enable health care providers to make informed decisions about resource allocation. Qualitative research can be conducted to further examine older adults’ perceptions of an in-hospital APEP intervention [86]. In large sample studies, sub-group or correlation analyses might be used to assess the association between the effect of the APEP intervention on the primary outcome and LOS, as well as age, baseline mobility, primary diagnosis, multimorbidity, or frailty [35]. To reach the sample size of 124 participants, the main study might be performed as a multi-centre study to address recruitment issues observed in a single hospital.

## Conclusions

This pilot RCT provides evidence that the proposed study protocol for a phase III trial to determine the effectiveness of an APEP intervention in acute geriatric care is feasible. Delivering the APEP intervention in older acute medical patients in the German GFK context seems safe and feasible, with the potential to induce small to moderate effects on mobility capacity. The results hint at a possible dose-response relation between exercise and the improvement of mobility. While the magnitude of the observed effects reaches clinical relevance for only some participants, effect sizes may become larger once the additional exercise training is being kept up for a longer time in other healthcare contexts. We consider a larger main study to examine the short- and long-term impact of the intervention on functional and hospital outcomes, in conjunction with a cost effectiveness analysis. Based on the improvements in mobility capacity (DEMMI), a total sample size of 124 participants should be included in the main study.

## Additional files

Additional file 1: Floor and ceiling effects of outcome measures. (PDF 277 kb)

Additional file 2: Histogram of the duration of all 90 APEP sessions performed in the trial. (PDF 271 kb)

## Data Availability

Data can be obtained from the corresponding author upon reasonable request.

## Abbreviations

ADL: Activities of daily living
APEP: Augmented prescribed exercise program
DEMMI: De Morton Mobility Index
DRG: Diagnosis Related Groups
ES: Effect size
FAC: Functional Ambulation Categories
FES-I: Falls Efficacy Scale-International
GFK: Geriatrische Frührehabilitative Komplexbehandlung
LOS: Length of stay
MMSE: Mini Mental State Examination
RCT: Randomized controlled trial
TUG: Timed up and go test
