# Supplementary material for: An augmented prescribed exercise program (APEP) to improve mobility of older acute medical patients – a randomized, controlled pilot and feasibility trial
Source: BMC Geriatr. 2019 Aug 30;19:240. doi: 10.1186/s12877-019-1246-4 (PMC6716827; doi:10.1186/s12877-019-1246-4)
Supplement: Supplementary file 1 — Floor and ceiling effects of outcome measures. (PDF 277 kb) [file 12877_2019_1246_MOESM1_ESM.pdf]

Additional file 1: Floor and ceiling effects of outcome measures

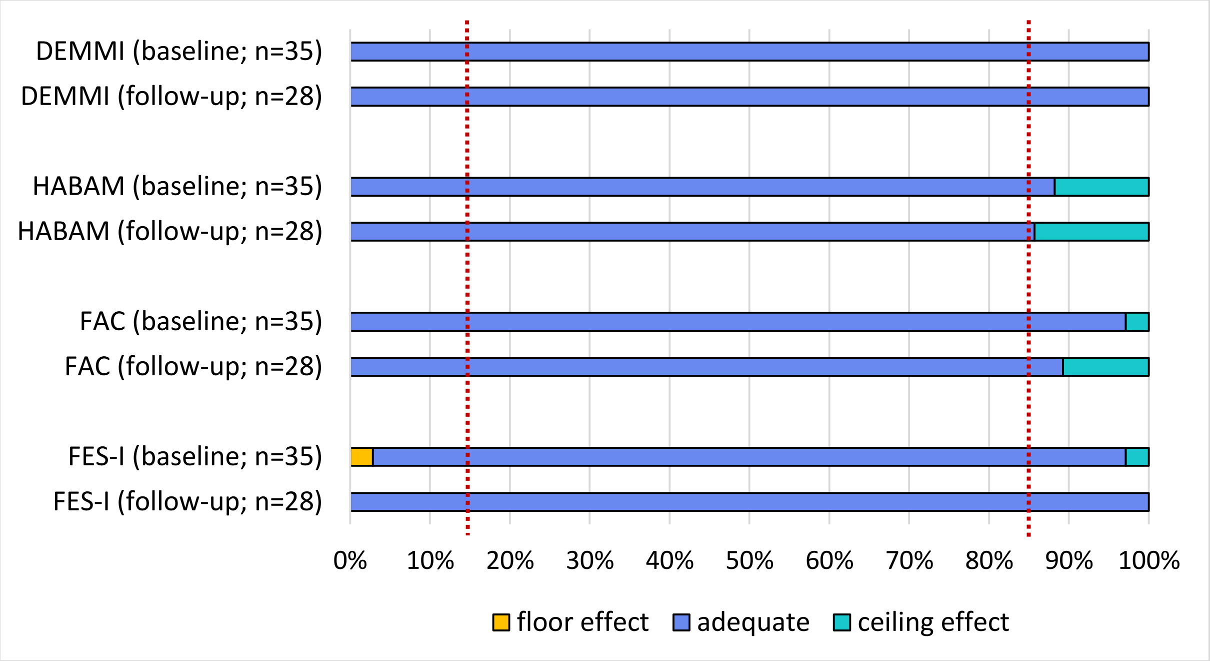

Dotted red lines indicate the threshold for a floor or ceiling effect of ≥15%.

Abbreviations: DEMMI = de Morton Mobility Index; HABAM = Hierarchical Assessment of Balance and Mobility; FAC = Functional Ambulation Categories; FES-I = Falls Efficacy Scale – International
