# Supplementary material for: An augmented prescribed exercise program (APEP) to improve mobility of older acute medical patients – a randomized, controlled pilot and feasibility trial
Source: BMC Geriatr. 2019 Aug 30;19:240. doi: 10.1186/s12877-019-1246-4 (PMC6716827; doi:10.1186/s12877-019-1246-4)
Supplement: Supplementary file 2 — Histogram of the duration of all 90 APEP sessions performed in the trial. (PDF 271 kb) [file 12877_2019_1246_MOESM2_ESM.pdf]

Additional file 2: Histogram of the duration of all 90 APEP sessions performed in the trial

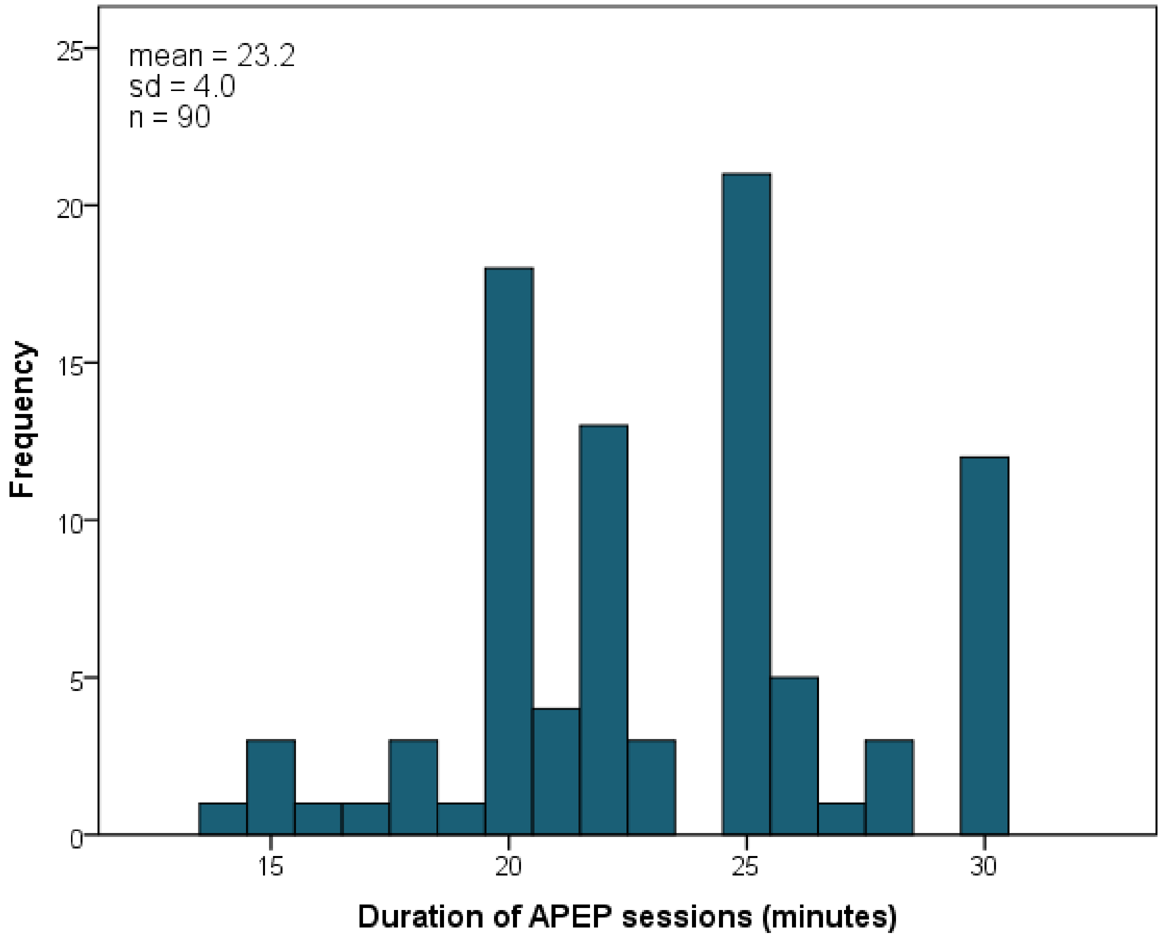

Abbreviations: sd = standard deviation; n = number; APEP = augmented prescribed exercise program
